# Intraspecific variation in metal tolerance modulate competition between two marine diatoms

**DOI:** 10.1038/s41396-021-01092-9

**Published:** 2021-08-26

**Authors:** Björn Andersson, Anna Godhe, Helena L. Filipsson, Linda Zetterholm, Lars Edler, Olof Berglund, Karin Rengefors

**Affiliations:** 1 Department of Marine Sciences, University of Gothenburg, Göteborg, Sweden; 2 Department of Geology, Lund University, Lund, Sweden; 3 Doktorsg. 9d, Weaq Lab, Ängelholm, Sweden; 4 Department of Biology, Lund University, Lund, Sweden

**Keywords:** Water microbiology, Microbial ecology, Microbial ecology

## Abstract

Despite widespread metal pollution of coastal ecosystems, little is known of its effect on marine phytoplankton. We designed a co-cultivation experiment to test if toxic dose–response relationships can be used to predict the competitive outcome of two species under metal stress. Specifically, we took into account intraspecific strain variation and selection. We used 72 h dose–response relationships to model how silver (Ag), cadmium (Cd), and copper (Cu) affect both intraspecific strain selection and competition between taxa in two marine diatoms (*Skeletonema marinoi* and *Thalassiosira baltica*). The models were validated against 10-day co-culture experiments, using four strains per species. In the control treatment, we could predict the outcome using strain-specific growth rates, suggesting low levels of competitive interactions between the species. Our models correctly predicted which species would gain a competitive advantage under toxic stress. However, the absolute inhibition levels were confounded by the development of chronic toxic stress, resulting in a higher long-term inhibition by Cd and Cu. We failed to detect species differences in average Cu tolerance, but the model accounting for strain selection accurately predicted a competitive advantage for *T. baltica*. Our findings demonstrate the importance of incorporating multiple strains when determining traits and when performing microbial competition experiments.

## Introduction

The world’s oceans are increasingly impacted by human activities causing climate change, eutrophication, and pollution [1]. Fitness of marine organisms can be directly affected by these environmental changes or indirectly through effects on competitors at the same trophic level, resource availability, and predation. Organisms may also evolve to compensate for fitness loss. Ecological and evolutionary effects on slow-growing organisms take time to develop, but microbial communities respond rapidly to environmental perturbation. Under toxic stress, sensitive microbial species are rapidly displaced by more tolerant ones [2–4], and in polluted soils and ponds, this generally reduces both diversity and ecosystem functionality [5–7]. Yet, our understanding of the structural effects of toxic compounds on marine phytoplankton communities is limited, despite their global ecological importance [8, 9].

On a global scale, the Baltic Sea is amongst the most perturbed marine systems. This sea currently experiences ocean acidification, warming, eutrophication, hypoxia, and pollution, which are projected to affect other coastal areas in the future [10]. Amongst other problems, this has led to bioaccumulation of heavy metals and organic pollutants in higher trophic levels of the marine food chain, but less is known about the effects of pollutants (macro-nutrients excluded) on the Baltic Sea microbial plankton community [11]. In seas and coastal waters, ambient pollution of copper (Cu), cadmium (Cd), and silver (Ag) may have structural effects on marine phytoplankton communities, although this has primarily been observed through the exclusion of highly sensitive cyanobacteria species [12–14]. In contrast, marine diatoms are considered to be more resilient to heavy metals [15], although observations are heavily biased towards model species in culture, and little is known about intraspecific variation. However, we recently observed that individual strains of a diatom population of *Skeletonema marinoi* Sarno and Zingone inhabiting the Cu mining-affected inlet of Gåsfjärden, on the Swedish Baltic Sea coast, displayed elevated tolerance to Cu (as well as towards several other metals), compared to a non-polluted reference population (Gropviken [16]). Based on silica frustules preserved in the Gåsfjärden sediment, it also appears that *S. marinoi* was severely depressed in relative abundance during the peak mining activity (circa 1600 to 1920 CE), and have recolonized the inlet since [17]. Changes in phytoplankton community composition have effects on higher trophic levels due to selective feeding mechanisms by zooplankton [18], and these observations suggest that the composition of costal diatoms in the Baltic Sea may be affected by metal pollution.

Variance in metal toxicity among marine phytoplankton species has mainly been characterized in the form of acute dose–response curves (72–96 h exposure) for a limited number of cultured model strains (e.g., [15, 19]). In contrast, traits such as light and thermal tolerance, *p*CO_2_ responses, nutrient uptake kinetics, and predator defenses, have been more widely characterized and found to vary significantly not only between phytoplankton taxa [20–22], but also among strains within local populations of species [23–26]. A potential limitation of these observations is that the majority are based on culture experiments on single strains in mono-culture (e.g., [24, 27–29]), which often fails to predict outcomes of co-cultivation experiments because of species or strain interactions in the shared aqueous environment [30–33]. Two well-known examples of such interactions are allelopathic chemical production [34] and luxury consumptions of nutrients [35]. Consequently, there is a need to further evaluate strain-based phenotypic observations in more complex biological settings.

We designed a co-cultivation experiment to test if strain-specific toxic dose–response relationships [36] can be used to construct deterministic models of how well *S. marinoi* compete with a related species (*Thalassiosira baltica* [Grunow] Ostenfeld) under metal stress (Ag, Cd, and Cu). To this end we used strain specific growth rates and inhibition coefficients to predict how selection on strains (four per species) modulates competition. The model was challenged against empirical observations during 10 days (10–20 generations) of semicontinuous co-cultivation. Similar models have been created to explore how differences in ecological traits such as temperature, nutrients, diversity, predatory defense, and light responses, structure phytoplankton communities [21, 22, 37–40]. However, to our knowledge, our model is the first that predicts how toxic responses modulate both strain selection and competition. We were especially interested in resolving if Cu toxicity affects fitness negatively in *S. marinoi*, since this provides a plausible ecological mechanism explaining the demographic patterns observed at the mining exposed Gåsfjärden population [16, 17] of the Baltic Sea. We chose to contrast *S. marinoi* with *T. baltica* since these two species comprise about 50% of the standing diatom biomass in the Baltic Sea and generally overlap temporally in abundance, suggesting that they are both ecologically important and natural competitors (Supplementary Fig. S1). By comparing the model’s outcome against the co-cultivation experiment, we explored if competitive interactions, strain selection, acclimatization [41, 42], or development of chronic stress [43] are important processes modulating species competition under toxic stress.

## Method

### Sampling sites, growth media, and physiological measurements

**Fig. 1:**
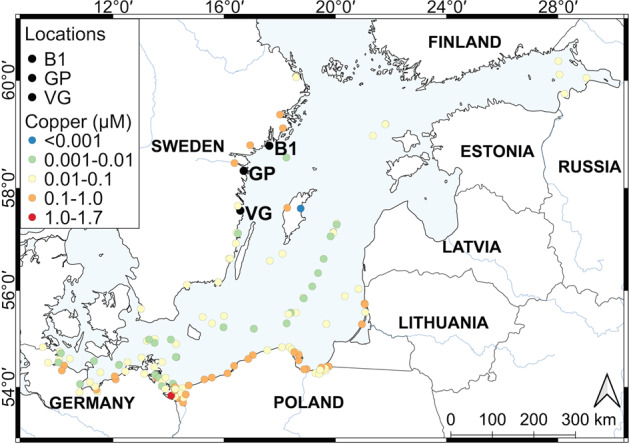
Map of the southern Baltic Sea showing sampling locations and monitoring data of Copper concentrations. Locations are Askö (B1: phytoplankton monitoring data), Gropviken (GP: retrieval site of strains used in this study), and Gåsfjärden (VG: copper mining site with elevated metal tolerance amongst *S. marinoi* strains compared with GP [16]). Colored dots show reported monitoring data of surface water concentrations of copper from ICES, IVL, and SLU databases. Note that for sites with multiple measurements only the maximum value is shown (see Appendix A for full data and method description).

Four strains each of non-axenic *S. marinoi* and *T. baltica* were isolated as single chains/cells from Gropviken (GP; 58°19.92′N 16°42.35′E) sediment resting stages as described previously [44]. We chose to conduct the competition experiment on strains from our previous reference site (GP), rather than the mining-affected site Gåsfjärden (VG), to assess tolerance differences in the more pristine of the two populations (Fig. 1). Experiments were performed at 16 °C under 180 μmol photons m^–2^ s^–1^ of photosynthetically active radiation (PAR): at 12:12 h light–dark cycles. Light was supplied by LUMILUXV Cool daylight L 36 W/865 light bars (OSRAM, Munich, Germany) and PAR was measured using a LI-COR^®^ LI-1400 light-meter with a scalar US-SQS/L detector (Walz, Effeltrich, Germany). In addition to the laboratory experiment, we analyzed monitoring data on Cu and Cd concentration and the seasonal phytoplankton bloom dynamics of a coastal site of the Baltic Sea, with specific emphasis on *S. marinoi* and *T. baltica* bloom dynamics (Supplementary Appendix A, C).

Site-specific seawater (salinity 7) was sterile-filtered (Sarstedt’s [Helsingborg, Sweden] 0.2 μm polyethersulfone membrane filter) and amended with f/2 concentrations of inorganic nutrients [45], with addition of 106 μM SiO_2_. In the f/2 media iron is chelated with 11.5 μM EDTA, which prevents iron precipitation but also reduces metal toxicity [46]. In the experiments, we subjected cultures to the average EC50 (effective concentration inhibiting growth rate by 50%) determined across all eight strains in Andersson et al. [16] which corresponded to 5.5, 9.7, and 0.046 μM, for Cd(II), Cu(II), and Ag(I), respectively (Cd, Cu and Ag, from here on). Metal amendment was done with acidified (pH 2), 100 μM stock-metal solution to growth media three hrs (±30 min) prior to the addition of cells. The absolute concentration of Cd, Ag, and Cu in stocks was quantified as described in [16] and chemicals were of analytical grade from Sigma Aldrich (St. Louis, MO, U.S.).

Culture biomass was routinely measured using in vivo chlorophyll *a* (chl *a*) fluorescence (RFU [relative fluorescence units]) using a Varioscan Flash Multimode Reader (ThermoScientific, Waltham, MA, U.S.), as described in [24]. RFU was measured midway into the light cycle (±1 h), and in the mono-strain cultures RFU measurements were used to calculate growth rates and inhibition coefficients (Eqs. 1, 2 below). During co-cultivation, 1 mL samples were preserved with 5 μL acidified Lugol’s solution, and microscopic observations were made using an Axiovert 135, Zeiss. Two hundred cells per species and samples were counted, or the total abundances in 1 mL if the densities were too low. Geometrical sizes of cells were computed based on dimensions of 20 cells per sample (×400 magnification) according to [47]. For the two diatom species in this study, RFU is most closely linked to biomass expressed as surface area [16] and we choose to utilize this unit to describe relative abundances and to compute species-specific growth rates under co-cultivation.

Pulse amplitude modulation (PAM) fluorometry was used to assess the physiological effect of metal treatment on the photosynthetic capacity of the cultures. The advantage of PAM fluorometry, in contrast to growth rate estimates, is that it can detect physiological stress instantaneously, without relying on two or more measurements over time. A Phyto-PAM [48] was used to measure temporal changes in the maximum quantum yield of photosynthesis (F_V_/F_M_). We used the 650 nm excitation light channel, and set the measurement frequency to 16 Hz to minimize actinic light effects in dark samples (<2 μmol photons m^–2^ s^–1^). Samples (1.5 mL) were dark-acclimatized for 10 min, to relax any quenching of chl *a* fluorescence and establish F_0_, after which a 200 ms long, 10,000 μmol photons m^–2^ s^–1^ strong, saturating pulse was used to elicit F_M_. F_V_/F_M_ was computed as (F_M_ − F_0_)/F_M_.

### Modeling outcome of selection under metal stress

The most widely used proxy for fitness in microalgae is exponential growth rate under a specific environmental condition. Exponential growth is also a condition that can be maintained and validated in an experimental setting [49], and it is the state of growth phytoplankton experience prior to the onset of nutrient depletion [39], once grazing mortality is corrected for [50]. Specific growth rates (μ) were computed using naturally log-transformed increments in density:

1 $$\upmu = {{{{{\mathrm{LN}}}}}}\left( {{{{{\mathrm{D}}}}}}_{{{{{{\mathrm{t}}}}}}1}/{{{{{\mathrm{D}}}}}}_{{{{{{\mathrm{t}}}}}}0} \right)/\left( {{{{{{\mathrm{t}}}}}}}_1 - {{{{{{\mathrm{t}}}}}}}_0 \right)$$

where D_t1_ and D_t0_ corresponds to the final and initial culture density and t_1_ − t_0_ the time difference between the measurements in days.

We used standardized acute 72 h toxic dose–response relationships to predict strain specific inhibition of growth rates by each metal [36]. Relationships for Ag, Cd, and Cu, (as well as Co, Ni, Pb, and Zn) were generated for the eight strains. A detailed description of the method can be found in [16]. From this data we extracted standardized (0–1) inhibition coefficients for the specific concentration of the metal assayed (I_MC[72 h]_), which combined with strain-specific growth rate estimates, can predict growth rate under the assay conditions:

2 $$\upmu_{MC} =\upmu - ({{{{{\mathrm{I}}}}}}_{MC( 72\,h)} \times \upmu )$$

For a specific strain, μ_MC_ corresponds to the growth rate under exposure to the metal M at concentration C. Long-term deviations between observed data and the prediction from Eq. (2) can be used to infer if acclimatization (Eq. (2) will underestimate growth rates) or chronic toxic effects (Eq. (2) will overestimate growth rates), are important long-term processes. By using μ_MC_ and rearranging Eq. (1) we made a deterministic model of how metal stress affects growth of a culture with an initial density of D_t0_ as a function of time (t):

3 $${{{{{\mathrm{D}}}}}}_{t} = {{{{{\mathrm{D}}}}}}_{t0} \times e^{{({{\upmu}_{{{{{{\mathrm{MC}}}}}}}} \times t)}}$$

The model can be extended to include species competition and intraspecific selection, i.e., a ‘Competition-Selection model’, by assuming that the individual species/strains do not modulate their own or other strains/species growth when co-cultivated (hereafter referred to as competitive interaction). When describing outcomes of competition and selection, we used relative density (RD) of species/strain *i* at time *t* with respect to the total number of species/strain *i-j* [25], which assuming that the start densities of all strains are the same, can be predicted as:

4 $${{{{{\mathrm{RD}}}}}}_{(i)t} = { e^{({\upmu} _{{{{{{\mathrm{MC}}}}}} i} \times t)}/{ \mathop{\sum}\limits_{{i - j}} {e^{({\upmu}_{{{{{{\mathrm{MC}}}}}}} \times t)}}}}$$

From this derivation we predicted the evolutionary response of the populations’ growth rate in response to selection by summarizing the individual contribution of all strains (μ_i-j_) to the population (μ_Pop_), given their predicted relative density at a given time (t).

5 $${\upmu} _{(Pop)t} = \mathop{\sum}\limits_{i - j} ({{{{{{{\mathrm{RD}}}}}}}}_{t} \times {\upmu})$$

For Eqs. (4), (5) to be valid we make two important assumptions. First, we assume indefinite, nutrient replete, exponential growth, which can only be achieved using a low density semicontinuous, or turbidostatic, cultivation approach [49]. Secondly, the Competition-Selection model assumes no competitive interaction that modulates growth rates (i.e., competitive interaction) or the toxicity of metals. As a contrast to the Competition-Selection model, we also predicted the competitive outcome on a Strain-by-Strain basis (16 predictions based on a *T. balticaS. marinoi* strain matrix), which illustrates how individual strain variability in tolerance influences the competitive outcome.

### Competition experiment

A schematic design of the competition experiment is shown in Fig. S2 and described in detail in Appendix B. Briefly, the experiment strived to subject the co-cultures (four strains each of *S. marinoi* and *T. baltica*) to Ag, Cd, and Cu stress, corresponding to the average EC50 amongst all eight strains. Nutrient replete, exponential growth was maintained through semicontinuous cultivation with serial dilution every 3 to 4 days according to [49]. To avoid nutrient limitation the maximum density was maintained below 30 mm^3^ biovolume cells L^–1^ media [16]. We monitored culture responses until one species was diluted to extinction in the control (defined as <1 cell mL^−1^), which took 10 days. Microscopy observations were done in connection with dilution on days 0, 4, 7, and 10. In parallel with the start of the competition experiment, we also made growth rate observations and metal inhibition measurements of the strains in mono-cultures (*N* = 2) in 1 mL of each treatment media (24-well microplates, Polystyren, Falcon).

### Statistical analyses and model implementation

Statistical analyses and model predictions were computed in R v. 3.6.2 [51]. Dose–response relationships and predictions of inhibition coefficients were generated using the drc package v. 3.0-1 [52] with Type 1 Weibull function [W1.2] for Cd and Type 2 Weibull [W2.2] for Ag and Cu [16]. Plotting and Model fittings were done in the package ggplot2 v. 3.2.1 [53]. Species differences in average growth and inhibition were tested using Welch *t* test, which does not assume homogeneous variances, using square-root transformed data. Differences in variance of traits between the species was assessed using the F-test. Paired *t* tests were used to test if individual strains varied in inhibition responses between the dose–response based prediction and the mono-culture observations. Linear mixed effects (LME) models were used to analyze the outcome of competition experiments and to test if growth rate, inhibition, and F_V_/F_M_ changes significantly between species, metals, and over time (fixed factors), using the nmle package [54] version 3.1-149. Replicate bottles were treated as random factors to account for pseudo-replication. For significant factors, post hoc tests were performed on hypothesis related comparison, not in a full factorial design, using pairwise Students *t* tests with Bonferroni corrected *p* values to account for multiple testing. The datasets generated during the study are available in the Dryad repository (doi.org/10.5061/dryad.280gb5mq9) and the Competition-Selection models code can be accessed through Git-Hub (https://github.com/Bearstar85/Selection_Competition_2021).

## Results

### Strain variability in growth and metal inhibition

**Fig. 2:**
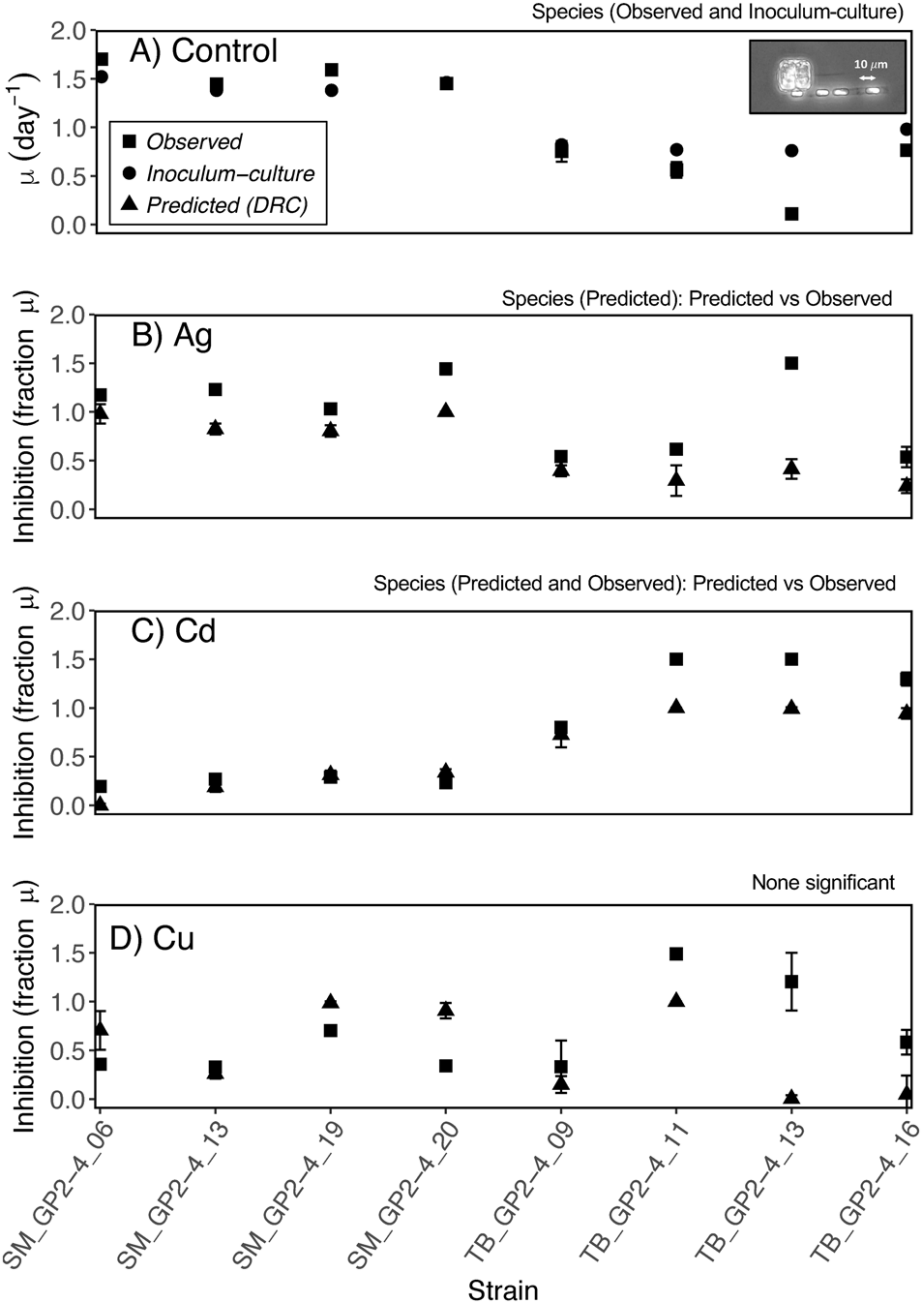
Strain variability in model parameters of *S. marinoi* (SM: smaller chain forming cells) and *T. baltica* (TB: larger solitary cell). (**A**) specific growth rate without toxic stress, and inhibition of growth rates when exposed to (**B**) silver [0.046 μM], (**C**) cadmium [5.51 μM], and (**D**) copper [9.73 μM]. Squares indicate observations in mono-cultures and triangles the inhibition that was predicted using dose–response (DRC) measurements from [16]. Circles in (**A**) show inoculum-culture growth rate, which was observed in the sub-culturing step prior to seeding the Competition experiment. Confidence intervals for Observed data show range (*N* = 2), and for the Predicted the model estimates with 95% confidence interval. When error bars are not visible, they are confined within the symbols. Significant differences (*p* < 0.05) are indicated on top of each panel based on Welsh *t* test (betweent Species) or paired *t* test (between Predicted vs. Observed inhibition estimates). Data were square-root transformed for statistical testing.

To validate the accuracy of the dose–response relationships, we contrasted dose–response predicted and observed inhibition on a strain basis (Fig. 2). In mono-culture, *S. marinoi* had an intrinsically higher growth rate than *T. baltica* under our assay conditions (Fig. 2A). For Ag, three *T. baltica* strains exhibited a stronger relative inhibition in the current assay than predicted (range 0.54–1.5 compared with 0.24–0.4, Fig. 2B). The fourth strain of *T. baltica* (TB_GP2-4_13) suffered a drastic decline in mono-culture growth rate and also became more sensitive to Ag, Cd, and Cu than predictions from the dose-response data. All four *S. marinoi* strains were less inhibited by Cd than *T. baltica*, and the strain prediction from previous data closely matched the observed inhibition, except that three *T. baltica* strains experienced negative growth (Inhibition >1), which the function cannot predict (Fig. 2C, Table S1). For Cu, we observed large and overlapping inter- and intraspecific variability in monoculture inhibition (Fig. 2D), and the species’ averages did not differ statistically using either assay approach.

### Modeling the outcome of competition and selection under metal stress

To predict the outcome of competition between the two species with potential for intraspecific selection, we constructed a Competition-Selection model that gave strains unique fitness features based on the predicted inhibition coefficients (Eqs. (4), (5)). In the Competition-Selection model it is not only the average, but also the variance between strains of a species that determine the competitive outcome. *T. baltica* had more variance in both basic growth rate (coefficient of variance [C.V.] 0.56 versus 0.08, F-test, *p* = 0.02) and, with the exception of predictions for Cd, trends of higher C.V. in all inhibition observations (F-test, *p* = 0.06 for Ag predicted, Ag observed, and Cd observed: Table S1).

The Competition-Selection model predicted that it would take 4 to 5 days in the control treatment for *S. marinoi* to account for >99% of the total biomass (Fig. S3A), due to its higher growth rate (average 1.55 ± 0.12, compared with 0.55 ± 0.31 day^–1^ for *T. baltica*). The Competition-Selection model also predicted that *S. marinoi* strain SM_GP2-4_06, with a growth rate of 1.75 ± 0.07 day^–1^, would account for 65% of the total *S. marinoi* biomass by day 10 (Fig. S3A). Under Ag stress, the Competition-Selection model predicted that *T. baltica* would rise close to competitive exclusion after 10 days (>99% of biomass) with an advantage for strain TB_GP2-4_09 and TB_GP2-4_16 (Fig. S3B). For Cd, *T. baltica* was predicted to be suppressed more rapidly than in the control, and that SM_GP2-4_06 would dominate the *S. marinoi* population (Fig. S3C), driven by both comparatively high growth rate and low Cd inhibition (Fig. 2C and Table S1). However, the dose-response curve of SM_GP2-4_06 displayed a pronounced degree of hormesis (stimulation of growth) at low Cd concentration (Fig. S4), which caused an underestimated predicted inhibition value, compared with the mono-culture observation (Table S1). Under Cu stress the Competition-Selection model suggested that *S. marinoi* still holds a small competitive advantage over *T. baltica*, but that a higher tolerance in two *T. baltica* strains slows down the competitive exclusion process (Fig. S3D).

### Competition without stress

**Fig. 3:**
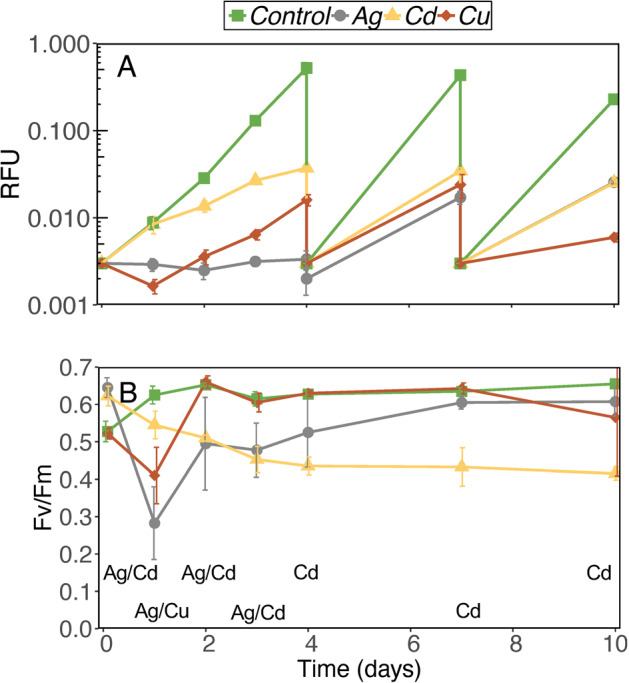
Changes in community (*S. marinoi* and *T. baltica* strains) density (A) measured as relative chlorophyll *a* fluorescence units (RFU) and maximum quantum yield of photosystem II (B) during the competition experiment. The community was semicontinuously diluted to 0.003 RFU before the end of exponential growth which occurs at 0.5 RFU in *S. marinoi* (which dominated the control) and 0.08 in *T. baltica*. Linear mixed effects models of F_V_/F_M_ identified significant differences between Metals (F_(3,12)_, *p* < 0.0001), Time (F_(6,72)_, *p* < 0.0001) and an interaction Metal × Time (F_(18,72)_, *p* < 0.0001). Metal names at the bottom of (**B**) indicate statistical differences relative to the control, based on post hoc test (*p* < 0.05). Error bars show S.D. (*N* = 4), and when error bars are not visible, they are confined within the symbols.

To validate the predictions from the Competition-Selection model we performed a competition experiment where all eight strains were mixed at equal RFU densities and subjected to Ag, Cd, or Cu stress, and a control treatment. For clarity, we have chosen to structure the results of the competition experiment individually for the four treatments. The changes in community density (RFU), and F_V_/F_M_, over the course of the experiment are shown in Fig. 3.

**Fig. 4:**
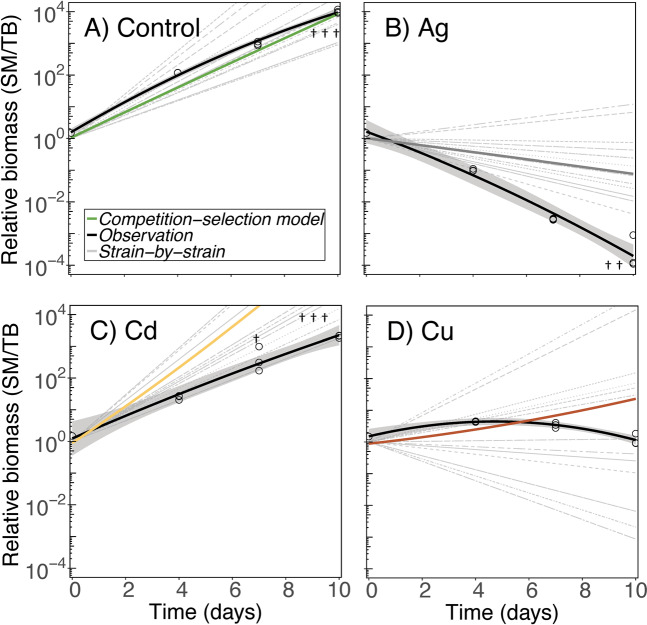
Observed and modeled changes in relative biomass (based on cellular surface area) of *S. marinoi* (SM) over *T. baltica* (TB) during the competition experiment. (**A**) shows control conditions, (**B**) silver stress, (**C**) cadmium stress, and (**D**) copper stress. Open circles correspond to observational microscopy data (*N* = 3), which have been fitted with a second order polynomial function, and 95% confidence intervals (black solid lines). When a species became extinct it was represented as the lowest detection limit (i.e., 1 cell mL^−1^) and such replicates are flagged with a single cross in the figure. Gray thin lines show modeled outcome of competition between individual strains in a factorial design (4 × 4 strains of each species). The Competition-Selection model (solid-colored lines) utilizes parameters of all eight strains simultaneously and allows for intraspecific selection. The model input parameters are shown in Table S1.

**Fig. 5:**
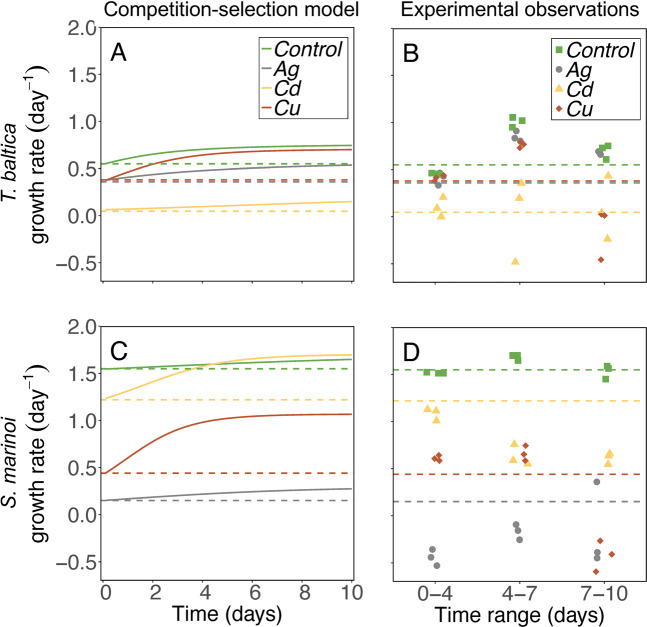
The Competition-Selection model prediction and experimental observations of changes in growth rate under co-cultivation. Solid lines in (**A**, **C**) show the dose-response predicted modeled outcome and (**A**, **B**) the observed changes of species-specific growth rate during the competition experiment. As a point of reference, the species averages are shown as dashed lines across both panels. The upper panel of graphs (**A**, **B**) corresponds to *T. baltica*, and the lower (**C**, **D**) to *S. marinoi*. The Competition-Selection model assumes no interaction between species or strain and no acclimatization or development of chronic toxic effects, only selection based on variability in inhibition predictions from dose–response curves (**A** and **C**). Observation data show specific growth rate observations computed based on microscopy observations on days 0, 4, 7, and 10 (*N* = 3) of the competition experiment (**B** and **D**). Note that statistical tests have been performed on inhibition data computed from growth rates as shown in (**B** and **D**) (see Table 1).

In the control treatment, the relative biomass between the two species changed as predicted from the Competition-Selection model during the 10 days (Fig. 4A). As expected from strain selection, growth rates also increased over time in both species and reached a peak at rates close to model predictions (Fig. 5). In the semicontinuous growth mode, culture densities oscillated between 0.4 and 30 mm^3^ biovolume L^−1^ media, incorporating the densities range observed during peak bloom conditions (Fig. S1). These results show that, although the two species were allowed to physically interact during co-cultivation, this did not modulate the growth rate of either species under nutrient replete conditions and realistic cell densities.

### Competition under silver stress

Based on RFU the Ag treatment had an immediate and severe detrimental effect on co-culture growth during the first 4 days. After this period growth improved, albeit at a slower rate than in the control (Fig. 3A). The F_V_/F_M_ showed a similar pattern of stress follow by recovery, and decreased significantly to 0.3 after 24 h, and then made a gradual recovery until day 4, when it could no longer be statistically differentiated from the control (Fig. 3B).

Based on both the Competition-Selection model and all Strain-by-Strain predictions, Ag stress was expected to consistently modulate competition in favor of *T. baltica*. This did happen, but all predictions underestimated the observed rate (Fig. 4B). One contributing factor was that the Ag Competition-Selection model utilized standardized inhibition coefficients and cannot predict negative growth (inhibition >1), which for *S. marinoi*, averaged 1.2 across the 10 days (Table 1). The Competition-Selection models predicted an increase in *T. baltica* growth rate under Ag stress, which was realized in the experiment (Fig. 5C, D). Inhibition could not be statistically ascertained compared to the control (average inhibition 0.095 post hoc test, *p* > 0.68, Table 1), which was lower than the strain specific observation (range 0.24–0.4 [predicted] and 0.54–1.5 [observed]; Table S2).

**Table 1:** Relative (0–1) inhibition of growth rate during the competition experiment.

	*Skeletonema marinoi*				*Thalassiosira baltica*			
Inhibition	Control	Ag*	Cd*	Cu	Control	Ag*	Cd*	Cu
Day 0–4	0 (0.004)	1.29 (0.057)	0.29 (0.043)	0.6 (0.019)	0 (0.012)	0.1 (0.15)	0.78 (0.23)	0.1 (0.068)
Day 4–7	0 (0.018)	1.1 (0.048)	0.62 (0.066)*	0.61 (0.048)	0 (0.053)	0.16 (0.054)	0.98 (0.44)	0.24 (0.03)
Day 7–10	0 (0.048)	1.1 (0.29)	0.6 (0.039)*	1.3 (0.11)*	0 (0.11)	0.025 (0.025)	0.89 (0.48)	1.2 (0.39)*
Average	0	1.2	0.5	0.84	0	0.095	0.88	0.51

Estimates of inhibition are based on microscopy observations on day 0, 4, 7, and 10, and the means (S.D.) are shown (*N* = 3). Linear mixed effects models identified all three factors (Metal, Time, and Species) as significant (all *p* < 0.0001) and also interactions between Species × Metal (F_(3,40)_, *p* < 0.0001) and Metal × Time (F_(6,40)_, *p* < 0.0001), but not the Species × Time (F_(2,40)_, *p* = 0.73). Asterisk and bold-formatted metals (Ag, Cd, and Cu) therefore indicate post hoc significant (*p* > 0.05) differences between the two species without considering Time effects. Asterisk and bold-formatted inhibition numbers indicate significant Time effect for Metals (compared with days 0–4).

### Competition under cadmium stress

In contrast to Ag, Cd had no effect on either RFU or F_V_/F_M_ during the first 24 h exposure. After 24 h both growth and F_V_/F_M_ declined, and by day 4 and beyond the F_V_/F_M_ stabilized at 0.45, compared with 0.65 in the control (Fig. 3). Cd exposure also induced morphological changes in both species. The cytoplasm of many *T. baltica* cells became darker under the light microscope, a sign of resting stage formation. Other *T. baltica* cells became elongated and on day 7 the average diameter:length ratio was 0.7 (±0.3) compared with 1.2 (±0.2) at day 0. Conversely, many *S. marinoi* cells became shorter and rounder with time, and the average diameter:length ratio increased from 0.4 (±0.1) to 0.5 (±0.2).

The Competition-Selection model predicted that Cd stress would speed up the increase in relative biomass of *S. marinoi*, but this did not occur (Fig. 4C). Instead, a chronic toxic response developed in *S. marinoi* manifested as a decrease in growth rate over time (Fig. 5D). However, the outcome was still competitive exclusion of *T. baltica* within 10 days of co-cultivation.

### Competition under copper stress

The Cu treatment caused an irregular temporal response, with a decrease in RFU and F_V_/F_M_ during 24 h post inoculation, after which net positive growth resumed (Fig. 3). By day 2, the F_V_/F_M_ had recovered to the control baseline of 0.65. This could be interpreted as an acclimatization response, but in a follow-up experiment we show that this was because the toxicity of Cu decreases for at least five hrs after it has been added to media (Fig. S5A), and cells were added after three hrs while Cu appeared to still be equilibrating with the media. No apparent changes in morphology compared with the control were observed in either species.

In the three separate experiments (dose–response relationship, mono-cultures, and during competition), the species differences in Cu tolerance could not be distinguished by comparing the average growth rate between the two species, using traditional *t*- or post hoc tests (Tables S1,  1). However, the trend of variance in tolerance was higher in *T. baltica*, not only for Cu, but across all metals (Table S1). In the competition experiment, Cu stress favored the *T. baltica* population, while the 16 Strain-by-Strain predictions resulted in a broad spectrum of variable competitive outcomes (Fig. 4D). Overall, the Competition-Selection model came close to predicting changes in relative biomass of the two species under co-cultivation (Fig. 4D), even though chronic toxic effects set in after day 7, and growth rates of both species collapsed <0 (Fig. 5B, D).

We conducted a database search for metal concentrations in seawater to test if, where, and when, Cu may exert toxic effect on Baltic Sea diatoms (Appendix C). To enable a reasonable comparison between the observed inhibitory data of this study and the environmental samples, we removed EDTA from the f/2 media and gathered complementary dose–response curves. As expected, this lowered the EC05 (effective concentration inhibiting growth rate by 5%) in *S. marinoi* from 5.3 to 0.26 μM (Fig. S5B). We used this lower observation as an indicator of the concentration at which Cu pollution may start exerting ecological and evolutionary effects on diatoms in the Baltic Sea. The majority of observations in the Baltic Sea databases were within the 0.01–0.1 μM range, but 0.4% exceeded the threshold of 0.26 μM (Fig. S6A). Out of 141 sample locations in the Baltic Sea, the >0.26 μM observations were distributed across nine locations (Fig. S6B). All sites were coastal (Sweden, Poland, Lithuania, and Germany) and close to (<50 km) or within major cities, river mouths, bays, or marinas (Fig. 1). There was high temporal variance in Cu concentrations, indicating that high Cu loading occurs in pulses. Seven of the nine highest recorded values were in spring (Jan–May: Table S2), coinciding with the timing of the spring diatom bloom.

## Discussion

### Lack of competitive interaction in co-culture

Whether or not phytoplankton community interactions can be modeled based on mono-culture trait observations is contested [30–33, 55], and likely taxon-specific. Deviations from expected growth patterns under co-cultivations have been attributed to differences in luxury consumption of nutrients [35], negative allelopathic chemical production [34], allelopathic stimulation of growth [30, 56], physical cell-cell interactions [57], and extracellularly mediated programmed cell death at high densities [58]. In contrast, we largely succeeded in modeling the outcome of the co-cultivation experiment using individual strain traits, suggesting low levels of modulated growth due to competitive interactions between *T. baltica* and *S. marinoi*, at ecologically relevant cell densities.

A prerequisite for our model was the maintenance of nutrient replete and thereby exponential growth. If unaccounted for, nutrient limitation can be a major confounding parameter in experiments [49, 59]. Inarguably, nutrient limitation is an ecologically important condition and we agree that it would most likely modulate the competitive outcome both by favoring the species with highest affinity for the limiting nutrient [60] or causing increased toxic sensitivity [16, 61, 62]. Yet, building a model and designing an experiment that accounts for these effects, would require accounting for both nutrient availability and assimilation. This involves measuring strain specific nutrient uptake kinetics, as well as interactions between nutrient limitation and toxicity, and employing a chemostat culturing approach to maintain balanced nutrient-limited growth [49]. Hence, further research is needed to explore how nutrient limitation modulate competition under toxic metal stress.

### Variability in metal tolerance between strains and species

Due to their large population sizes and high genetic diversity, phytoplankton have been suggested to evolve rapidly [63, 64]. In accordance, we observed that predicted increase in species growth rate via strain selection was realized in the Control, Ag, and Cu treatments, highlighting that evolution of metal tolerance through selection on standing genetic diversity can occur on short timescales in diatoms (only 10–20 generations passed in the 10-day experiment). Other artificial evolution experiments, mainly focused on climate-related conditions such as warming and elevated *p*CO_2_, have shown that there is potential for other phytoplankton species to evolve through selection on standing genetic diversity amongst strains [23, 55, 65]. Population wide studies have been performed for a wide range of phytoplankton traits, with the most common interpretation of the results being to compare averages between populations, and not evolutionary potential (but see [25, 66]). Our findings stress the importance of interpreting intraspecific variation of populations through evolutionary modeling, and not statistical tests that compare changes in the mean (such as *t*-test and post hoc test associated with ANOVA or LME analysis). To this end, our modeling may be modified to include more species or strains, other types of response curves, or fluctuations in selection pressures along the response gradient.

Arguably, our study was limited in scope to only four strains of each species, far from the expected population size of phytoplankton (thousands of clones per bloom [67]). Hence, we are likely capturing only a fraction of the evolutionary potential of the two populations. Nevertheless, our observations regarding competitive response to Cd and Ag appear robust enough to represent the species, given the lack of overlap in tolerance towards these two metals. Regarding Cu, we cannot rule out that random sampling effects confound our competitive prediction. However, we observed more variance in both growth and inhibition traits (trend in seven out of eight traits) in *T. baltica* compared to *S. marinoi*. Therefore, we expect that adding more strains to the population would lead to the discovery of more tolerant *T. baltica* compared to *S. marinoi* strains, in line with our competitive prediction for Cu. Nevertheless, future evolutionary studies should strive to encompassed larger populations to gain a better understand the role of intraspecific diversity in shifting ecological interactions between diatom species. This can be achieved through either natural community mesocosm experiment or via high-throughput phenotyping combined with artificial evolution experiments.

### Variability in modes of toxicity among metals

The three metals caused distinct patterns in modulation of species competition. Most strikingly, Cd completely inhibited *T. baltica* while *S. marinoi* continued to grow, while for Ag, the reverse effect was observed (Fig. 5). This is surprising since selection experiments on natural communities of microbes often show that selection for one metal causes co-selection for tolerance to other metals [5, 68, 69]. Cd, unlike Cu and Ag, is mutagenic to bacteria [70] and it is possible *T. baltica* is more sensitive to DNA damage than *S. marinoi*. The silica frustule changes we observed in both species under Cd stress may be deformities caused by disruption of the cell-cycle or silicification processes, which could have a mutagenic origin [43]. The slow development of Cd toxicity is in line with such an indirect mode of inhibition. Another possibility is that there was positive selection for certain morphotypes, a phenomenon that was recently observed in *Phaeodactylum tricornutum*, were an oval morphotype is more resistant to Cd due to differences in cell wall structural properties [71]. However, in our experiment fitness was expected to recover over time if there is strong selection for strain morphotypes, but this was not observed.

An expected challenge to our modeling effort was that metal toxicity would change over time, either due to chronic stress or physiological acclimatization. In our experiments, chronic stress was the more dominating of the two processes. The slow development of chronic toxicity towards Cu and Cd provides a challenge for utilizing acute dose-response data to predict long-term inhibition patterns. However, changes in species’ biomass ratios could still be accurately predicted for Cu (Fig. 4A), since chronic toxicity affected both species similarly (Fig. 5). On the other hand, both growth rate and biomass ratios predictions were affected by chronic toxicity under Cd stress, since inhibition levels increased only in *S. marinoi*. We suggest that future modeling could be improved through parametrization of the time-dependent (chronic) inhibition component. Another strategy could be to pre-acclimatize the cultures to toxic stress before experiments [15], although it is questionable if this is ecologically relevant for marine phytoplankton that generally experience pulses of toxicity associated with point sources of pollution from land run-off, ships, or marinas (Table S2: [11, 72]).

Phytoplankton have a remarkable acclimatization potential to abiotic stressors such as temperature, acidification, nutrient limitation, and irradiance changes [66, 73–75]. Our experiment, in line with previous observations [76], suggests that for toxic metal stress, the acclimatization processes are less important than the development of chronic stress. Undoubtedly, the cells still undergo some acclimatization process to metal stress, but our results suggest that this process may be rapid enough to be largely incorporated in the acute 72 h dose–responses. Metal exposure has been shown to trigger rapid (12–24 h) reductions in phytoplankton transporter activity, production of phytochelators, and upregulation of oxidative stress responses in other microalgae species [41, 77, 78], which supports this conclusion.

### Copper toxicity in the Baltic Sea

A relevant question to our laboratory study is whether Baltic Sea diatoms ever experience toxic Ag, Cd, and Cu concentrations in their ambient environment? In our Baltic Sea database search, we found no evidence Cd would reach concentrations that cause toxicity, and no data on Ag pollution. In contrast, we found reports of comparatively high Cu concentrations. Strict comparisons between environmental metal concentrations and culture predicted inhibition are difficult due to the strong chelation of Cu ions by compounds that are selectively removed and amended to the growth media. Further complicating factors such as nutrient limitation, stress from multiple metals, dissolved and particulate organic material, changes in salinity and inorganic ligand concentrations, also interact to modulate the toxicity experience by phytoplankton [11, 12, 16, 46, 61, 70]. When we removed EDTA from the media, we observed Cu toxicity (EC05) at concentrations of 0.26 μM, which was lower than observational data at certain locations with high anthropogenic loading (Figs. 1, S6). Although this does not provide conclusive evidence, it suggests that Cu may have structural effects on local diatom populations in the Baltic Sea.

In our laboratory experiments, we observed that Cu stress causes a long-term reduction in *S. marinoi* fitness in competition with *T. baltica*. Although it is possible that other diatom species are more or less sensitive to Cu, these two species comprise about 50% of the standing diatom biomass in Baltic Sea spring blooms (Fig. S1). We therefore expect that *S. marinoi* would suffer a competitive disadvantage at toxic Cu concentrations. It may explain why *S. marinoi* went locally extinct at the copper mining exposed Gåsfjärden inlet from the 1600s to the late 1900s [17], and has evolved Cu tolerance during later re-colonization [16]. Although we cannot provide further evidence of drivers behind such historical events, our observations here warrant further field research into marine areas that experience high Cu loading (e.g., Baltic Sea locations identified in Table S2). Such studies could provide direct evidence for evolutionary and ecological effects of metal pollution on coastal phytoplankton communities.

## Conclusions

We have shown that different metal stress can modulate diatom species competition in opposite directions (Cd and Ag), suggesting that the mechanism conferring tolerance differs between both species and metals. Based on *S. marinoi* and *T. baltica*, it appears possible to utilize mono-culture fitness measurements to model diatom species competition, opening an avenue to make ecological and evolutionary predictions based on toxicological laboratory observation. However, relying on single microalgal strains, or population averages, to make species generalizations can lead to incorrect and largely random ecological conclusions. Instead, it is necessary to quantify and account for intraspecific trait variance to successfully predict how toxic stress affects microbial systems. A formidable future challenge is how to sufficiently account for standing genetic variation within the enormous population sizes of eukaryotic phytoplankton.

## Supplementary information

Supplemental material
