# Supplementary material for: Intraspecific variation in metal tolerance modulate competition between two marine diatoms
Source: ISME J. 2021 Aug 26;16(2):511–20. doi: 10.1038/s41396-021-01092-9 (PMC8776739; doi:10.1038/s41396-021-01092-9)
Supplement: Supplementary file 1 — Supplemental material [file 41396_2021_1092_MOESM1_ESM.docx]

**Supplemental material: Intra-specific variation in metal tolerance modulate competition between two marine diatoms**

**Björn Andersson, Anna Godhe, Helena L. Filipsson, Linda Zetterholm, Lars Edler, Olof Berglund^,^ and Karin Rengefors**

*Corresponding author: [bjorn.andersson@marine.gu.se](mailto:bjorn.andersson@marine.gu.se)

**Table of content:**

**Appendix A: Monitoring data of phytoplankton (page 1-4)**

**Appendix B: Modeling and experimental procedure (page 5-10)**

**Appendix C: Monitoring data of heavy metals (page 11-15)**

**References (page 16)**

*Appendix A: Monitoring data of phytoplankton*

We used the phytoplankton monitoring data timeseries from Askö (station B1: 58° 48.11' N 17° 37.31' E) to investigate the degree of co-occurrence of *T. baltica* and *S. marinoi* in the coastal part of the Baltic Sea. This timeseries began in 1983 and samples (0–20 m depth integrated tub method) have been collected at approximately bi-weekly intervals with more frequent sampling during March and April (often weekly), and less frequently during the winter months (1–2 times per month, with missing data for some winter months in the 80’s and 90’s). We acquired the species or genus level data from the Swedish Meteorological Institute’s (SMHI) website (https://sharkweb.smhi.se, accessed 2019–06–05), and collapsed data from all taxa, save *S. marinoi* and *T. baltica*, into the major taxonomical groups: other diatoms, cyanobacteria, dinoflagellates, and ‘other microalgae’ consisting of algal species with primary phototrophic growth (e.g. small flagellates, chrysophytes, haptophytes, and chlorophytes) using Plankton Toolbox v 1.3.1 (1). To account for the large cell size difference between taxa, including *S. marinoi* and *T. baltica* (the latter is about 50–fold larger in terms of biovolume) we chose to analyze biovolume data. We noted that at low cellular densities, the limit of detection was significantly higher, in terms of biovolume, for *T. baltica*. It was therefore difficult to compare trends in the two species when zero observations have been made for *T. baltica*, but not *S. marinoi,* which occurs frequently in summer and fall.

The phenology of phytoplankton at Askö (B1) since it was in close proximity to our diatom sample site Gropviken (GP: Fig. 1), and the site has a long-running and high-resolution dataset. The main aim of this analysis was to identify the relative contribution and temporal overlap in the blooms of *S. marinoi* and *T. baltica*, which is shown for the last decade in Fig. S1A. During this period there is a clear pattern of overlap between the two species during the spring bloom. *S. marinoi* also occur in fall and summer most years, although this observation need to be cautiously interpreted since the detection limit for the significantly larger *T. baltica* is about 10 times higher than for *S. marinoi* (5×10^–4^ compared with 5×10^–5^ mm^3^ biovolume L^–1^ water due to differences in cell size) and abundances of *S. marinoi* is generally two to three orders of magnitude lower than T. baltica in spring (Fig. S1A). Once data was smoothened across the year 1983–2019, the trend of co-occurrence of the two species in the spring, along that of other diatoms, was obvious, while other diatom species dominate the fall bloom (Fig. S1B). The combined standing biomass of *S. marinoi* and *T. baltica* amounts to about 50% of the total standing biomass of pelagic diatoms at Askö (Fig. S1B), but with considerable year-to-year variability during the peak (Fig. S1C).

The seasonal oscillations in the whole phytoplankton community at Askö show the three characteristic phases of the Baltic Sea (Fig. S1B). Diatom dominate the spring bloom in February-April and may also contribute to the fall bloom in September-October. Dinoflagellates and other phototrophic taxa contribute to both blooms, but generally peak after the diatom bloom has ended. In the summer, when inorganic nitrogen concentrations limits growth, there are blooms of noxious nitrogen fixing cyanobacteria, mixotrophic dinoflagellate and nano-flagellates and other smaller microalgae taxa.


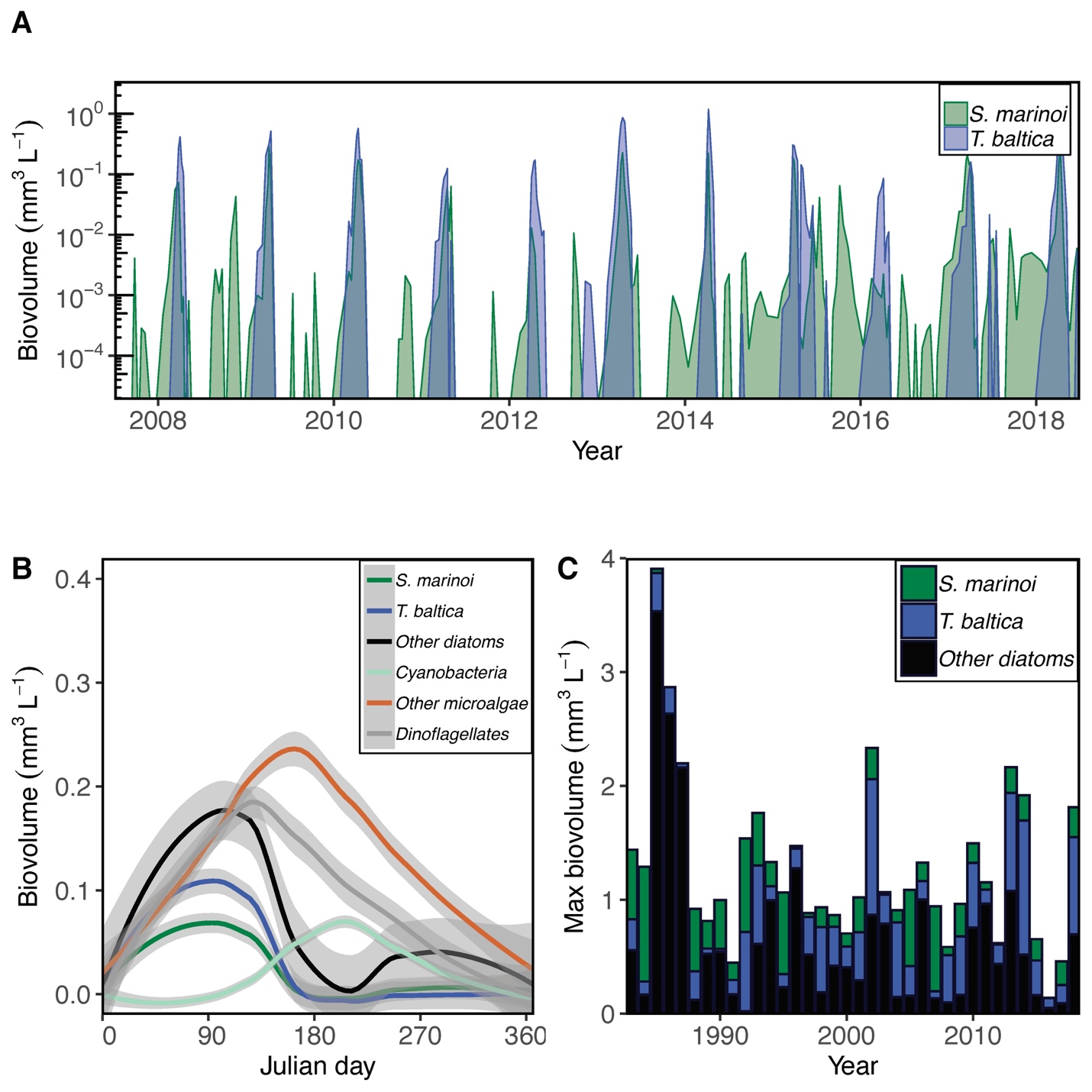


Fig. S1: Summary of phytoplankton monitoring data from Askö station B1. A) Fluctuations in biovolume of *S. marinoi* and *T. baltica* during the past decade (2008-2018). Values below lower limit of y-axis indicates no detectable cells, with the detection limit for *T. baltica* at about 5×10^-4^ and *S. marinoi* at 5×10^-5^ mm^3^ L^-1^. B) Seasonal changes in the total biomass of the phytoplankton community, with *S. marinoi* and *T. baltica* separated from the group ‘other diatoms’. Data is based on data across 1983 to 2018 with smoothed averages fitted using the *loess* equation in ggplot2. Gray areas correspond to 95% confidence intervals. C) Yearly variation in the maximum observed biovolume of diatoms.

*Appendix B: Modeling and experimental procedure*

|  | Control | | Ag** | | Cd* | | Cu^(ns)^ | |
| --- | --- | --- | --- | --- | --- | --- | --- | --- |
| Strain/Species | Growth rate inoculum-culture (day^-1^) | Growth rate (day^-1^ [Range]) | Predicted inhibition (Relative [95% conf.]) | Observed inhibition (Relative [Range]) | Predicted inhibition (Relative [95% conf.]) | Observed inhibition (Relative [Range]) | Predicted inhibition (Relative [95% conf.]) | Observed inhibition (Relative [Range]) |
| SM_GP2-4_06 | 1.52 | 1.7 (1.7–1.8) | 0.98 (0.88–1) | 1.17 (1.21–1.18) | 0.00023 (0–0.017) | 0.19 (0.18–0.2) | 0.7 (0.51–0.9) | 0.36 (0.32–0.4) |
| SM_GP2-4_13 | 1.38 | 1.44 (1.4–1.4) | 0.82 (0.77–0.88) | 1.23 (1.19–1.27) | 0.188 (0.14–0.24) | 0.27 (0.27–0.27) | 0.26 (0.21–0.31) | 0.33 (0.29–0.36) |
| SM_GP2-4_19 | 1.38 | 1.59 (1.6–1.6) | 0.8 (0.74–0.86) | 1.03 (1.04–1.02) | 0.31 (0.27–0.36) | 0.29 (0.26–0.32) | 0.99 (0.97–1) | 0.7 (0.68–0.72) |
| SM_GP2-4_20 | 1.46 | 1.45 (1.5–1.4) | 1 (1–1) | 1.44 (1.38–1.5) | 0.34 (0.31–0.37) | 0.23 (0.22–0.24) | 0.91 (0.83–0.98) | 0.34 (0.33–0.35) |
| TB_GP2-4_09 | 0.82 | 0.75 (0.65–0.86) | 0.4 (0.34–0.45) | 0.54 (0.49–0.59) | 0.72 (0.6–0.85) | 0.8 (0.77–0.83) | 0.15 (0.063–0.24) | 0.33 (0.06–0.6) |
| TB_GP2-4_11 | 0.77 | 0.56 (0.48–0.64) | 0.29 (0.14–0.45) | 0.62 (0.58–0.65) | 1 (1–1) | 1.5 (1.5–1.5) | 1 (1–1) | 1.5 (1.5–1.5) |
| TB_GP2-4_13 | 0.76 | 0.11 (0.061–0.16) | 0.41 (0.31–0.52) | 1.5 (1.5–1.5) | 1 (0.97–1) | 1.5 (1.5–1.5) | 0.0027 (0–0.04) | 1.5 (0.91–1.5) |
| TB_GP2-4_16 | 0.98 | 0.76 (0.75–0.78) | 0.24 (0.17–0.31) | 0.54 (0.64–0.43) | 0.94 (0.89–1) | 1.3 (1.22–1.36) | 0.048 (0–0.24) | 0.58 (0.46–0.71) |
| S. marinoi (C.V) | 1.44 (0.047)*** | 1.55 (0.08^†^)* | 0.9 (0.11)*** | 1.2 (0.12) | 0.21 (0.74)* | 0.25 (0.17)** | 0.71 (0.45) | 0.43 (0.42) |
| T. baltica (C.V) | 0.83 (0.12) | 0.55 (0.56) | 0.34 (0.25) | 0.8 (0.59) | 0.91 (0.14) | 1.27 (0.26) | 0.3 (1.6) | 0.98 (0.62) |

Table S1. Empirically derived parameters used in modeling outcome of competition experiment. Growth rate of inoculum-culture was collected based on density measurements three days before, and at the day the experiment was set up (N=1). Growth rates were collected for individual strains on 24 well plates in parallel with competition experiment (first 72-h, N=2). Dose-response curve predictions (N=24 total replicates across 6 concentrations) of inhibition was based on data collected in Andersson et al. (2020). Observed inhibition was measured in parallel with the start of the competition experiment for the first 72-h in 24-well plates using the same media as in the competition experiment (N=2). Note that dose-response curve predicted inhibition [I_MC(72h)_] is constrained between 0-1, while the observed inhibition was not capped until 1.5, which corresponded to the detection limit of the plate reader. All of the above-mentioned parameters uses density measurements based on relative chl *a* florescence units (RFU). A cross (†) symbol indicate a significant F-test, which suggest that the variance is different between the two species. Asterisks on metals indicates significant differences between the DRC predicted and parallel observation of inhibition based on paired *t*-test*.* Data was square-rot transformed for statistical tests. The species averages, with the coefficient of variance in brackets, is shown at the bottom with significant species differences in averages based on Welch *t*-test (without assumption of homogeneous variances). **p<0.05,* ***p<0.01,* ****p<0.001.*

**Competition experimental design**

To test if we could use the dose-response measurements to predict the outcome of long-term competition between under toxic stress, we designed the competition experiment (Fig. S2). The experiment strived to subject mixed communities (four strains each of *S. marinoi* and *T. baltica* from the GP site) to Ag, Cd and Cu stress, corresponding to on average 50% inhibition of growth rate amongst strains. We then employed a semi-continuous cultivation approach as described in detail by Andersson, Godhe (2), and also in (3), to maintain nutrient replete, exponential growth through serial dilutions every third day, and monitored culture responses until one species was diluted to extinction (defined as <1 cell mL^-1^).

We used *in vivo* relative Chl a fluorescence (RFU) to standardize the density of each of the four *S. marinoi* and *T. baltica* strains and mixed them at equal proportion for a final RFU of 0.003 in 50 mL of Cd, Cu and Ag prepared media, as well as a control with no metals added beyond the contents of the f/2 nutrients (N=4 experimental replicates per treatment). To avoid nutrient limitation the maximum density was maintained below 0.03 mm^3^ biovolume cells mL^–1^ media (RFU<0.5 when S. marinoi dominates, and <0.08 for *T. baltica* domination), after which inorganic carbon becomes limiting. On the start day of the competition experiment mono-strain cultures were concentrated in 50 mL Falcon tubes, using gentle centrifugation (5 min at × 1000 g), and washed twice in clean media to remove any extracellular compounds, which could influence metal bioavailability, or act as allelopathic chemicals. *T. baltica* is approximately 50 times larger than *S. marinoi* so for the two species this corresponded to a predicted start density of 60 and 400 cells mL^–1^, respectively. Given the culture volume, this translates to an estimated 5,000 cells of each *S. marinoi* strain, and 750 of each *T. baltica* strain included in the inoculum of each replicate.

In parallel with the mixed community treatment, we made growth rate and inhibition observations of the individual strains (N=2) in 1 mL of each treatment media on 24–well microplates (Polystyren, Falcon^TM^) with daily measurements of RFU over the course of seven day (without re-inoculations). This was done for two reasons: 1) to measure the absolute growth rate of each strain which could not be expected to remain stable since the earlier dose-response measurements because growth rate vary in diatoms as a function of cell size (4); 2) to test the reproducibility in predicted inhibition of specific strain and species from the dose-response measurements in Andersson, Godhe (2). The co-cultivation experiments were performed 2–3 months after isolation and generation of the dose-response measurements. In-between these experiments, cultures had been maintained at 10°C under 10 μmol photons m^–2^ s^–1^ PAR and a 12:12 hrs light-dark cycles, to minimize growth rates and potential for evolutionary responses to the laboratory environment (5). After 1 week of acclimatization, the experiment was performed at 16°C under 180 μmol photons m^–2^ s^–1^ PAR and 12:12 hrs light-dark cycles.


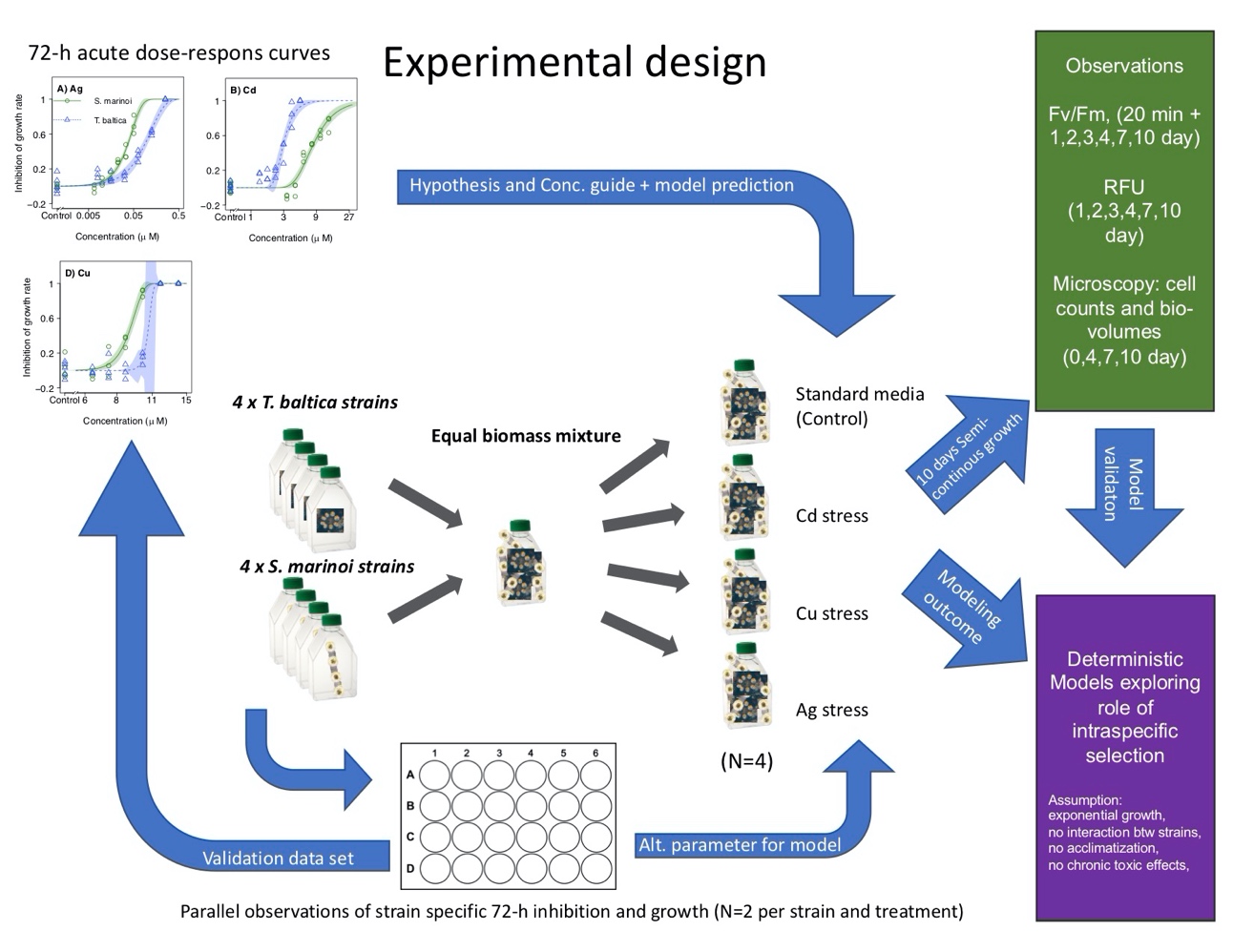


Fig. S2. Schematic illustration of the competition experimental design.

Fig. S3 Modeled outcome of competition on relative abundances of individual strains depending on strain-specific estimates of growth rate and inhibition by metals. Green shades correspond to *S. marinoi* and blue to *T. baltica* strains.


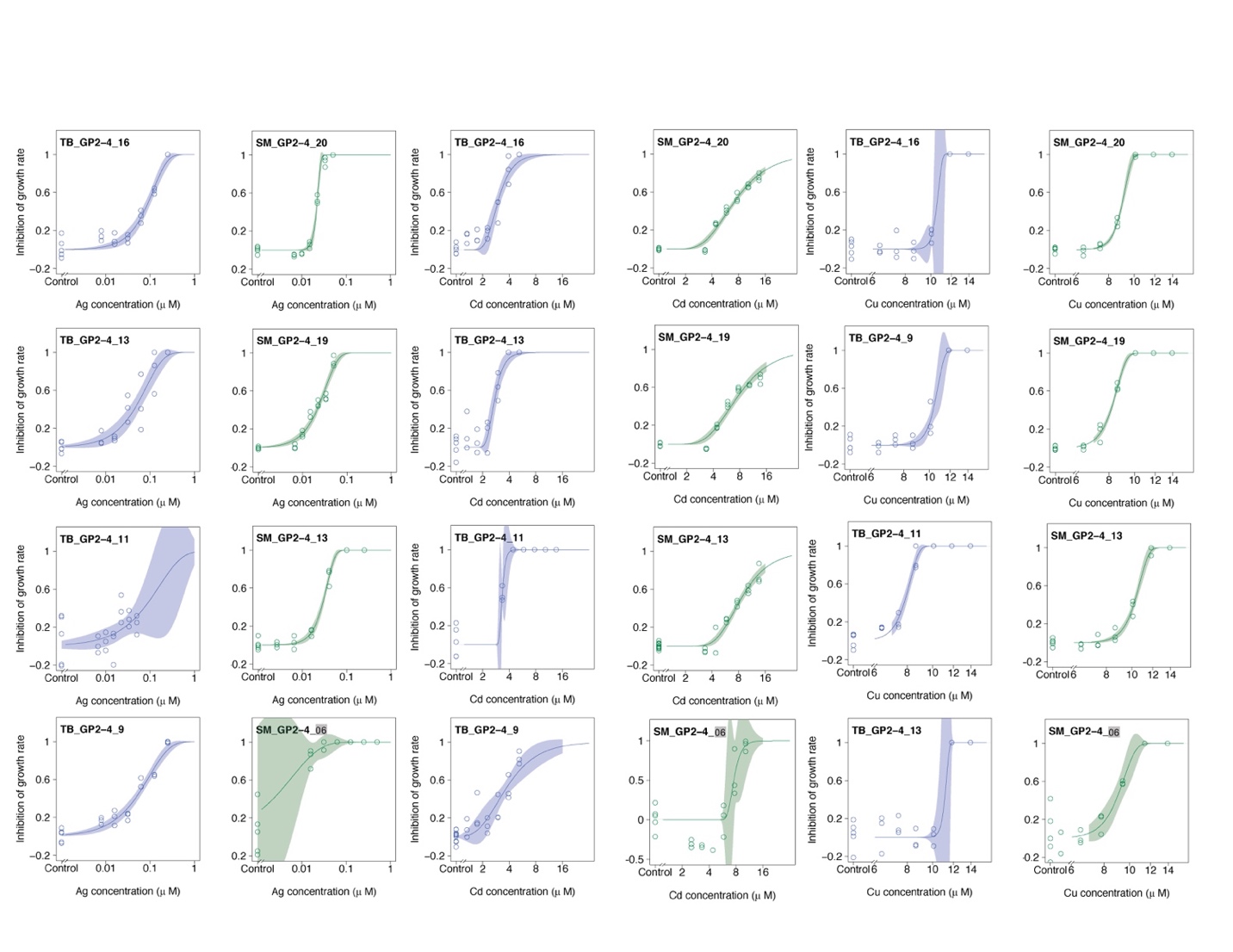
Fig. S4: Dose-response curves with 95% confidence intervals for all strains and metals used in this study (underlying data for predictions in Fig. 2 of the main manuscript). Open circles correspond to replicates (N=24 per metal and strain, and six dilution steps) of observational specific growth rate data, which have been standardized to inhibition through dividing by the mean of the control replicates as I=1-(μ_Observed_/μ_Mean_Control_). The Green curves are *S. marinoi* and Blue *T. baltica*. The metal is specified in the x-axis title. Note that the y-scale differs for Cd in SM_GP2-4_06 to accommodate the hormesis response, which is more pronounced than in the other *S. marioni* strains.

*Appendix C: Monitoring data of heavy metals*

We searched both Swedish and international databases for seawater measurements of heavy metals in the Baltic Sea, with the aim of identify regions with potentially diatom-toxic metal concentrations. We identified the International Council for the Exploration of the Sea (ICES) database as covering the largest area of the Baltic Sea, and containing both Cadmium (Cd) and Copper (Cd) measurements from seawater samples (https://ecosystemdata.ices.dk). However, the Swedish coast of the Baltic Sea was not represented in ICES data so in addition we searched Swedish databases and identified water measurements of Cu in the Swedish Environmental Research Institute (IVL: https://dvsb.ivl.se/dvss/DataSelect.aspx) and the Swedish University of Agricultural Sciences (SLU: https://miljodata.slu.sehttps://miljodata.slu.se) databases. We did not find any databases containing silver (Ag) data. Data from these three databases was downloaded (2020–06–23) and trimmed to only include water measurements from the Baltic Sea, and river mouths (<2 km upstream of the sea), which resulted in 122 (ICES), 7 (SLU), and 12 (IVL) locations. We made no distinction between filtered and unfiltered samples since this was infrequently reported. Since we were interested in identifying regions with potential toxic levels of metals, we choose to visualize the maximum values from sites with more than one observation. Results were visualized in QGis v. 3.10.

ICES database had 1966 observations of Cu in water and 2042 for Cd, after we omitted observations with data reported as ug/kg (286 observations), which were unreasonably high for water samples and presumably contained erroneously indexed sediment or biota samples. In the Swedish databases (IVL and SLU) we only identified Cu measurements in water samples. In the ICES data, the min, median, and max observations for Cu was 0, 0.013, and 1.7 μM, and for Cd 0, 0.00044 and 0.018 μM. Because of the comparatively low Cd concentrations, and the lack of data in IVL and SLU, we choose to focus the rest of our analysis on Cu. Cu data from the three databases originated from 141 localities, with an avarage of 16 observations per site. The highest observed value for each site is shown in Fig. 1 of the main manuscript.

To enable a reasonable comparison between the observed inhibitory data of this study and the environmental samples, we removed EDTA from the f/2 media and gathered contrasting dose-response measurements. This lowered the EC05 (effective concentration inhibition growth rate at 5%) in *S. marinoi* from 5.3 to 0.26 μM (Fig. S5B). We used this lower observation as an indicator of the concentration were Cu pollution could start excerting ecological and evolutionary effects on diatoms in the Baltic Sea. While the majority of observation in the Baltic Sea databases were within the 0.01-0.1 μM range (Fig. S6A), 0.4% were above the EC05 threshold (1% above the lower 95% confidence interval). Out of the 141 locations (2277 observation points), nine locations at some point in time experienced higher than EC05 concentrations (Fig. S6B). All sites where costal (Sweden, Polen, Lithania, and Germany) and close to (<50 km) or within mayor cities, river mouths, bays, or marinas (Fig. 1). In proximity of the bay of Gdynia, Kiel harbour, and Zalew Szczeciński Lagoon there were more than two locations with >0.26 μM Cu concentrations. With the exception of the two Polish sites 3ZP and 4ZP, which had median observations of 0.1 μM, compared with 0.02–0.03 for the other seven, these locations only experienced concentrations above 0.26 μM for one singel observation (Fig. S6). Seven of these highest recorded value were in spring (Jan–May: Table S2) coinciding with the spring diatom bloom. These results suggest that concentrations of Cu could have toxic effect on diatoms in the Baltic, primarily around areas of strong anthrophogenic loading.


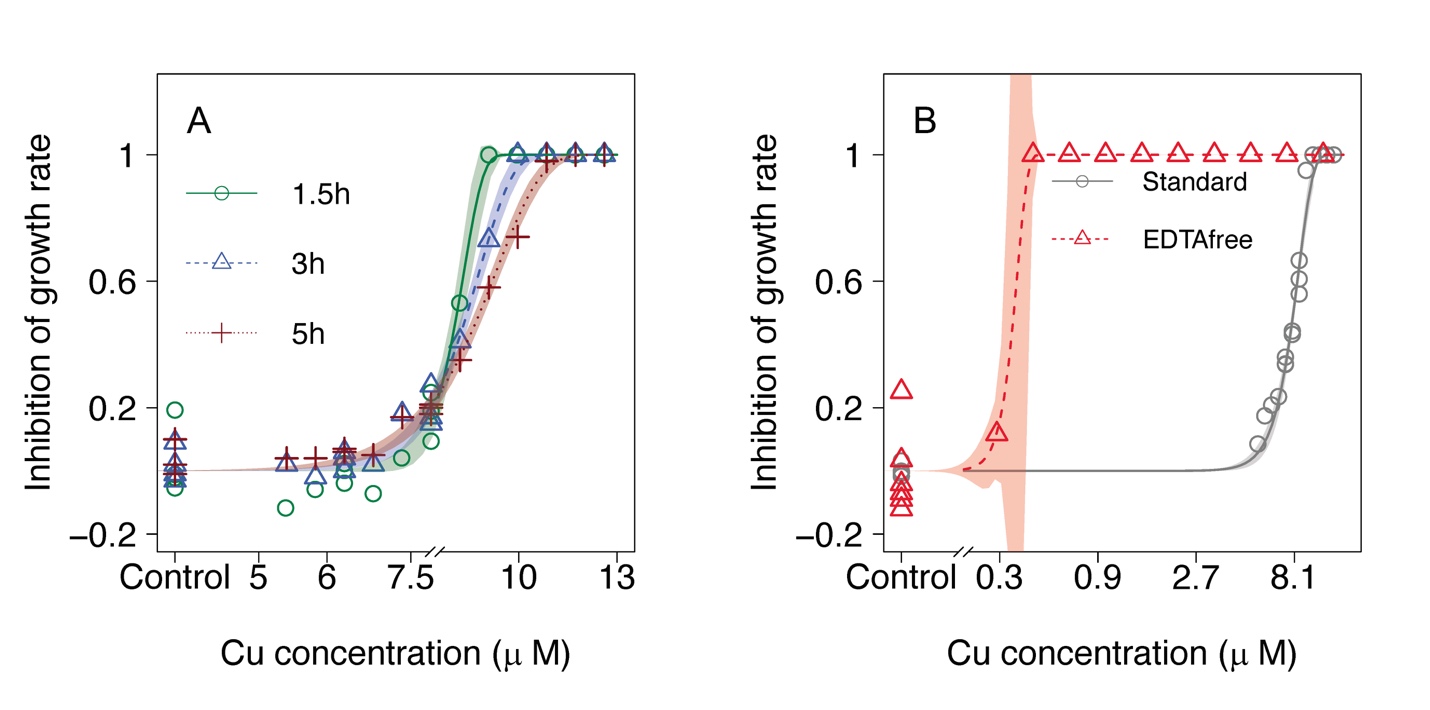
Fig. S5. Dose-response curves for copper in *S. marinoi* strain RO5AC. A) modulation of toxicity as an effect of changes in the time between mixing Cu with media (standard f/2 in all treatments) and addition of algal cells (1.5, 3, and 5 h wait period). Note that 3 ± 0.5 h wait time was used in the competition experiment. B) contrast in toxicity between standard f/2 media versus EDTA free media (with 3-h wait-time). The dilution series has been modified from Andersson et al. 2020 with 12 instead of 6 dilution steps and only replication around the expected EC05, EC50 and EC95, which improved the resolution significantly (contrast with Cu in Fig. S3). Note that the x-axis’s between A and B have different ranges, but both are logarithmic.

Fig. S6. Distribution of copper concentration observations. A) Cumulative probability of all copper concentration observations in Baltic Sea water (N=2277). Solid red vertical line at *S. marinoi* strain RO5AC EDTA-free EC05 (dashed line 95% confidence interval). B) Boxplot of Cu concentrations in the 9 Baltic Sea locations where water sample Cu concentrations exceed estimated EC05 of *S. marinoi*. All temporal observations are shown with median and 25 and 75 percentile in boxes, and whisker cover the range, with single points as outlier based on ggplot2 geom_boxplot function with default settings. See Table S2 for more details on these locations.

| Station | Data-base | Country | Location | Lat. (deg.dec N) | | Long. (deg.dec E) | | Median Cu (μM) | Range (μM) | Number of observations | Month max observation |
| --- | --- | --- | --- | --- | --- | --- | --- | --- | --- | --- | --- |
| 3ZP | ICES | Poland | 60 km north-east of Zalew Szczeciński Lagoon and Oder river mouth | | 54.113 | | 15.065 | 0.130 | 0.079–1.7 | 4 | May |
| 4ZP | ICES | Poland | 10 km south-west from 3ZP | | 54.167 | | 15.291 | 0.160 | 0.063–0.57 | 4 | May |
| K41 | ICES | Lithuania | Between the cites of Kalingrad and Klaipėda (ca 30 km from either) | | 55.312 | | 20.9567 | 0.018 | n.d.–0.31 | 80 | Feb |
| Linnéaholm | IVL | Sweden | Stockholms outer archiepelago | | 59.377 | | 18.011 | 0.390 | 0.39–0.39 | 1 | Jan |
| Nyköpingsån Spånga | SLU | Sweden | River mouth with marina located near the city Nyköping | | 58.813 | | 16.937 | 0.015 | 0.0054–0.55 | 50 | Jan |
| OM225103 | ICES | Germany | 1 km from port of Kiel, close to mouth of the Nord-Ostsee-Kanal | | 54.3538 | | 10.16467 | 0.024 | 0.011–0.49 | 18 | Jun |
| OMMVGB19 | ICES | Germany | Bay, 40 km north-west of Zalew Szczeciński Lagoon and Oder river mouth | | 54.2067 | | 13.5667 | 0.011 | 0.00079–0.62 | 59 | Aug |
| OMMVKHM | ICES | Germany | Inside Zalew Szczeciński Lagoon | | 53.825 | | 14.1 | 0.020 | 0.0031–1.4 | 115 | Apr |
| T18P | ICES | Poland | Bay of Gdansk | | 54.379 | | 18.878 | 0.044 | 0.024–0.71 | 6 | Jul |

Table S2. Summary of locations with Cu concentrations exceeding EC05 (effectiv concentration inhibition growth rate at 5%) of *S. marinoi* strain RO5AC, in f/2 media without EDTA (0.26 μM).

Refrences

1. Karlson B, Andreasson A, Johansen M, Mohlin M, Skjevik A, Strömberg P, editors. Plankton Toolbox–open source software making it easier to work with plankton data. In Proc 16th international conference on harmful algae, ed A L MacKenzie, 194–197 Cawthron Institute, Nelson, New Zealand and the International Society for the Study of Harmful Algae (ISSHA); 2016.

2. Andersson B, Godhe A, Filipsson HL, Rengefors K, Berglund O. Differences in metal tolerance among strains, populations, and species of marine diatoms-importance of exponential growth for quantification. Aquat Toxicol. 2020;226:105551.

3. MacIntyre HL, Cullen JJ. Using cultures to investigate the physiological ecology of microalgae. In Andersen, R A (Ed): Algal culturing techniques (Elsevier). 2005:287-326.

4. Von Dassow P, Chepurnov VA, Armbrust E. Relationships between growth rate, cell size, and induction of spermatogenesis in the centric diatom Thalassiosira weissflogii (Bacillariophyta). J Phycol. 2006;42(4):887-99.

5. Lakeman MB, von Dassow P, Cattolico RA. The strain concept in phytoplankton ecology. Harmful Algae. 2009;8(5):746-58.
